# Efficient Sparse Signal Transmission over a Lossy Link Using Compressive Sensing

**DOI:** 10.3390/s150819880

**Published:** 2015-08-13

**Authors:** Liantao Wu, Kai Yu, Dongyu Cao, Yuhen Hu, Zhi Wang

**Affiliations:** 1 State Key Laboratory of Industrial Control Technology, Zhejiang University, Zheda Road 38th, Hangzhou 310027, China; E-Mails: wuliantao@zju.edu.cn (L.W.); kaiyuzju@gmail.com (K.Y.); dongyu@zju.edu.cn (D.C.); 2 Department of Electrical and Computer Engineering, University of Wisconsin-Madison, Madison, WI 53706, USA; E-Mail: yhhu@wisc.edu

**Keywords:** lossy wireless link, compressive sensing, sparse signal transmission, packet length control

## Abstract

Reliable data transmission over lossy communication link is expensive due to overheads for error protection. For signals that have inherent sparse structures, compressive sensing (CS) is applied to facilitate efficient sparse signal transmissions over lossy communication links without data compression or error protection. The natural packet loss in the lossy link is modeled as a random sampling process of the transmitted data, and the original signal will be reconstructed from the lossy transmission results using the CS-based reconstruction method at the receiving end. The impacts of packet lengths on transmission efficiency under different channel conditions have been discussed, and interleaving is incorporated to mitigate the impact of burst data loss. Extensive simulations and experiments have been conducted and compared to the traditional automatic repeat request (ARQ) interpolation technique, and very favorable results have been observed in terms of both accuracy of the reconstructed signals and the transmission energy consumption. Furthermore, the packet length effect provides useful insights for using compressed sensing for efficient sparse signal transmission via lossy links.

## 1. Introduction

Wireless sensor networks (WSNs) have attracted much interest in both academia and industry due to their advantages of low cost, ease of deployment and ubiquitous applications, ranging from military surveillance to medical monitoring [1]. Reliable wireless communication is of paramount importance to facilitate data-centric WSN applications. However, low power wireless links often lead to a high data packet loss rate [2] due to factors like channel noise, multipath, asymmetric links, inadequate antenna orientation [3] and antenna height [4]. Data collected from large-scale WSN installations are often mired with missing or garbled data values, which significantly depreciates the potential usage of the data.

To deal with the high data packet loss rate, one may either transmit data only via high quality links in the connected region to ensure communication reliability; or invoke error-correcting measures, such as automatic repeat request (ARQ), in the transitional region to retransmit lost data packets (the communication area of a wireless link can be divided into three regions, which are called the connected region, the transitional region and the disconnected region). Using only high quality links in the connected region to transmit data implies shorter physical distance, which also leads to denser sensor node deployment or higher transmission power, *i.e*., shorter network lifespan. ARQ-based error correcting measures will increase transmission times and latency [5,6]. Both approaches are expensive and imperfect solutions.

There are also many schemes for improving the communication performance of wireless systems, such as transmitting power control, packet length control, forward error correction (FEC), *etc*. However, FEC and power control are energy-consuming schemes [7] and are unsuitable to be implemented in low power wireless sensor nodes [8]. The scheme presented in this work is a different asymmetric communication method that shifts the computation burden from the transmission end (low power sensor nodes) to the receiving end (more powerful fusion center).

Our approach leverages compressive sensing [9,10] at the receiving end to reconstruct the original sparse signal; even data packets are lost or damaged while transmitting in a lossy wireless link. In fact, with the careful packaging of the data using the appropriate packet lengths and interleaving scheme, the effect of transmission loss may be modeled as a random sampling process. As such, the original data may be reconstructed with a high probability.

The notion of incorporating compressive sensing with network data transmission was discussed in [11], where the authors proposed a distributed matched source-channel communication scheme by exploiting the spatial averaging property inherent in a multiple access channel. Numerous authors have reported approaches to combine compressive sensing with wireless communication by exploiting the inherent sparseness in signals, such as temperature, $CO_{2}$ emission, seismic signal [8], image [10] and acoustical signal [12].

This work is a substantial expansion of an earlier conference proceeding paper [12], where the notion of modeling lossy wireless links as a random sampling process in compressive sensing was first presented. In this paper, we provide a careful derivation to show how the packet loss in a lossy channel may be modeled as a random sampling process of the original signal. In addition, the intricate relations between communication parameters (e.g., bit error rate, packet error rate, packet lengths) and the quality of signal reconstruction (mutual coherence) are carefully investigated. To mitigate potential burst communication error, the interleaving scheme is incorporated at little computation and energy overhead at the transmission end. Moreover, a novel criterion, called the information acquisition rate (IAR), is proposed as a more direct and effective way to gauge the link quality rather than traditional quality metrics, such as the packet reception rate (PRR). We have conducted extensive simulation to validate the proposed scheme, and a field experiment with a wireless sensor network that implements this proposed scheme is also conducted. Both simulation and experimental results demonstrated superior performance.

The main contributions of this paper can be summarized as follows:

- We demonstrated the feasibility of incorporating compressive sensing as an error correction measure to facilitate communication over lossy wireless links.
- We propose a new cyber-physical measurement process model in applying compressive sensing where the packet loss in a wireless link is modeled as a random sampling process.
- We propose a novel wireless link performance metric, called the information acquisition rate, to measure the actual information content that is transferred. We show that this metric better reflects the actual performance of data transmission than the conventional data-oriented criteria, such as the packet reception rate.
- We established important relations between packet lengths and the mutual coherence, which is critical to the success of compressive sensing reconstruction.

The rest of this paper is organized as follows. Section 2 and Section 3 review the related work and compressive sensing (CS) theory, respectively. We present the proposed sparse signal transmission framework in Section 4. Packet length control for sparse signal transmission is shown in Section 5. Section 6 shows comparison simulations between the conventional ARQ interpolation technique and the CS-based method, as well as the packet length effect on sparse signal transmission. Experimental verification is presented in Section 7. Finally, Section 8 concludes this paper.

The following notation is used throughout the paper. Vectors and matrices are denoted by boldface lowercase and boldface uppercase letters, respectively. The identity matrix is denoted by **I**. For any matrix **A**, $A^{\prime }$ or $A^{T}$ refers to the matrix transpose; Tr(A) is the trace. The $l_{p}$ norm of a vector **x**, for 1≤p≤∞, is denoted $\left||x\right|{|}_{p}={\left(\sum_{i=1}^{N}\left|x\right|\right)}^{1/p}$, while $\left||x\right|{|}_{0}$ denotes the number of nonzero elements in **x**. The main notation in this paper is listed in Table 1.

## 2. Related Work

Information acquisition consists of data transmission and signal recovery. The most frequently-used approach for providing reliable data transmission over lossy links is to use the acknowledgment-based retransmission mechanism, at the cost of extra energy consumption and latency. Specifically, the energy cost to successfully transmit a packet over a lossy link is proportional to the number of transmissions (including the first transmission and subsequent retransmissions if necessary) for that packet. Furthermore, traditionally, transmission power is adjusted dynamically to overcome unreliability over lossy links in energy-constrained WSNs. The network node increases its transmission power to achieve immunity against link errors. Nevertheless, besides the extra energy consumed, [13] illuminates that it adversely affects the channel contention, network throughput and energy consumption at the network scale.

**Table 1:** Symbols and notations.

Symbol	Explanation
**x**	sampled signal, $x={\left[x_{1},...,x_{n}\right]}^{T}$
$\hat{x}$	reconstruction signal, $\hat{x}=\left({\hat{x}}_{1},{\hat{x}}_{2}...{\hat{x}}_{N}\right)$
*ε*	reconstruction error, $\varepsilon =||x-\hat{x}|{|}_{2}/\left||x\right|{|}_{2}$
*N*	dimension of sampled signal
*M*	dimension of projection (measurement) vector
**Ψ**	fast Fourier transform matrix, $\Psi \in R^{N\times N}$
**Φ**	projection matrix,$\Phi \in R^{M\times N}$
*α*	coefficients that represent **x** on the basis Ψ, $\alpha ={\left[\alpha_{1},...,\alpha_{N}\right]}^{T}$
*K*	nonzero coefficients in *α*
**A**	equivalent matrix, A=ΦΨ
**y**	measurement vector, y=Φx
μ(A)	mutual coherence
$\tilde{A}$	column-normalized version of **A**
**G**	$G={\tilde{A}}^{T}\tilde{A}$
$P_{prr}$	packet reception rate (PRR)
$P_{e}$	bit error rate (BER)
*L*	packet length
$L_{overhead}$	overhead length
$L_{payload}$	payload length
*n*	packet number $n=\frac{N}{L_{payload}}$

Other approaches try to study the link behavior for better understanding of the wireless communication characteristics and transmit data packets based on this to achieve better performance. The works in [5,6,14] utilize link burstiness to achieve better communication performance. The work in [5] presents a metric denoted *β* to measure this link burstiness. Measuring *β* allows one to reason about how long a protocol should pause after encountering a packet failure to reduce its transmission cost. The works in [6,14] characterize links by their maximum burst length and then adaptively allocate slots for reliable data transmission. However, link-aware methods always induce large transmission delay and extra energy cost, because of waiting for a good link; besides they are all data-centric methods, which must transmit almost all of the data sampled to sink node to get information detected.

Once the data packet has been received, the conventional interpolation technique is used for signal recovery. Despite much progress in the area of data interpolation, existing methods are suitable for only a few data loss condition, but cannot scale when the rate of data loss is high [15]. Furthermore, data fusion algorithms have always been adopted for denoising, overcoming environmental effects, *etc*. [16]. In practical WSNs applications, multi-sensor data fusion can also be applied at the fusion center for performance promotion.

Advances in compressive sensing [9,10] offer a promising approach for signal acquisition and recovery. One appealing feature of the CS theory is its ability to reconstruct the original high-dimensional, inherently sparse signal by using only very few low dimensional measurements, whichprovides a promising prospect for signal recovery. This attractive feature motivated a number of works applying CS to wireless communication.

The authors of [11] are the first to introduce compressed sensing into network applications. The authors propose a distributed matched source-channel communication scheme by exploiting the spatial averaging inherent in a multiple access channel. Furthermore, [11] has enlightened CS’ potential application in sensor networks. Since then, there have been many research works that have concentrated on combining CS with wireless communication for transmitting these sparse signals.

In the application layer, [15] proposed a CS algorithm to estimate the missing data based on the typical data loss pattern, and [7] introduced compressive sensing as a coding strategy that is applied in the application layer, which exploits redundancy in oversampling to facilitate compressive oversampling.

In the routing layer, [17,18] represent the first complete design to apply compressive sampling theory to sensor data gathering for large-scale wireless sensor networks. The proposed scheme can handle abnormal sensor readings gracefully, and its load balancing characteristic is capable of extending the lifetime of the entire sensor network. In [19], the effectiveness of data gathering in WSNs is improved by introducing an autoregressive model to assess inherent sparsity and to adjust the number of measurements accordingly.

In the medium access control (MAC) layer, [20] proposed compressive sensing MAC (CS-MAC) to exploit the sparse property of the sensing signal that, at a given time, only a few hosts are expected to access the channel for a data report. In CS-MAC, a central coordinator can recover a multitude of these received data in one decoding operation and then schedule multiple hosts accordingly. This result is incorporated in [21], which boosts multiple input multiple output (MIMO) capacity gain without requiring strict synchronization and coordination among distributed users. The work in [22] proposed a random access compressed sensing (RACS) scheme to analyze random access to the wireless channel. In RACS, a randomly-chosen subset of nodes participates in the sensing process and then randomly accesses the channel to report their sensing data. By using the node’s carefully-designed sensing probability, RACS ensures sufficiently many data packets to be received in spite of packet collision.

In the link layer, we are the first to combine CS with data loss via a lossy wireless link [12]. We modeled the packet loss during transmission as a random sampling process by utilizing knowledge of signal characteristics (sparse), then the sparse signal can be accurately transmitted through sufficient received data with lower energy consumption and latency.

In this paper, considering that packet length control is an easy to implement and efficient communication performance optimization method, we further investigate packet length effect on sparse signal transmission via a lossy link based on our former work [12]. Packet length control for sparse signal transmission is investigated, which inspires us to choose different optimal packet lengths to optimize different communication demands. Furthermore, for the traditional communication technique, data interleaving is adopted to optimize sparse signal transmission performance. Further details are presented in the following sections.

## 3. Compressive Sensing

Compressive sensing is an emerging novel signal acquisition and recovery method that has become a hot research topic recently; it provides an alternative to Shannon/Nyquist sampling when the signal under acquisition is known to be sparse or compressible [9].

### 3.1. Compressive Sensing Fundamentals

Compressive sensing includes three parts:

- Sparsity: Let $x={\left[x_{1},...,x_{n}\right]}^{T}$ represent the *N*-dimension original signal and $\Psi =[\psi_{1},...,\psi_{N}]$ be an orthogonal basis (dictionary), such that:
  (1) $$x=\sum_{i=1}^{N}\alpha_{i}\psi_{i}=\Psi \alpha$$
  where $\alpha ={\left[\alpha_{1},...,\alpha_{N}\right]}^{T}$ is the vector that represent **x** on the basis **Ψ**. The signal is sparse if most of the elements of *α* are zero or they can be discarded without much loss of information. Specifically, if there are K(K≪N) nonzero coefficients in *α*, the signal can be regarded as being *K*-sparse. In fact, sparse signals are rather ubiquitous, such as acoustic signal, temperature, the density of carbon dioxide and images, which allows compressive sensing to be applied in many far-reaching applications in WSNs [23].
- Incoherent measurement: For any *N*-dimensional signal x, its measurement y is taken as follows:
  (2) y=Φx=ΦΨα=Aα
  where $y\in R^{M}$ is the *M*-dimensional linear measurement data, $\Phi ={\left[\phi_{1},...,\phi_{M}\right]}^{T}$ is the M×N projection matrix and A=ΦΨ is the equivalent matrix. Compressive sensing theory requires the projection matrix **Φ** and the sparse dictionary to be as incoherent as possible, such that the samples add new information that is not already represented by the known basis **Ψ**. It has been proven [24] that **Φ** with i.i.d. Gaussian entries with zero mean and variance $\frac{1}{M}$ or binary matrices with independent entries taking values $\pm \frac{1}{\sqrt{M}}$ are largely incoherent with any fixed sparse dictionary **Ψ** with overwhelming probability as long as the measurement number conforms to the following condition, holding for some constant *c*:
  (3) M≥cKlog(N/K)
- Reconstruction algorithms: *K*-sparse x can be reconstructed by solving the $l_{0}$ norm [10] from y as follows:
  (4) $$\min||x|{|}_{0}\;\;\;s.t.\;\;\;\;\;\Phi \Psi x=y$$
  This optimization problem relies on an exhaustive search and is successful for all $x\in \sum_{K}$ when the matrix **Φ** has the sparse solution uniqueness property. However, this algorithm has combinatorial computational complexity [25]. An alternative to the above $l_{0}$ norm optimization is to use the $l_{1}$ norm. $l_{1}$ norm optimization is convex, which can be solved by using linear programming, and the global optimal solutions can be achieved. An alternative to optimization-based approaches is greedy algorithms for sparse signal recovery. These methods are iterative in nature and select columns of **Φ** according to their correlation with the measurements y determined by an appropriate inner product. A typical example is orthogonal matching pursuit algorithms (OMP), which we use in our framework due to the low complexity. Still, the computational complexity of this solution is greater than that of traditional decoding and data interpolation, but CS shifts this burden to the base station, which we assume to have a significantly higher energy budget than the ordinary sensor nodes. Considering that the data have noise in practice, basis pursuit de-noising (BPDN) (also called LASSO) is adopted to minimize the usual sum of square errors, which can be formatted as a $l_{1}$-penalty least squares estimate problem:
  (5) $$\underset{\alpha }{\arg\min}||y-{\Phi \Psi \alpha ||}_{2}^{2}+\lambda ||\alpha |{|}_{1}$$
  It should be noted that some authors reserve this term for the related optimization problem, with a bound on the sum of the absolute value:
  (6) $$\min_{\alpha }\left||\alpha \right|{|}_{1}\;s.t.||y-{\Phi \Psi \alpha ||}_{2}^{2}\leq \eta$$

### 3.2. Compressive Sensing Applications

In this part, we introduce several examples of combining CS with practical systems and applications, such as wireless channel estimation, network tomography and magnetic resonance (MR) image reconstruction. In channel estimation, a wideband signal reveals the actual response of the wireless channel, which has a discrete nature consisting of multipath components; while the response is smoothed out for narrowband communication systems, which makes the impulse response of the wireless channel tend to be sparse for a larger bandwidth [26]. In link delay estimation of network tomography, only a limited number of links have large delays in typical networks. If considering a vector composed of delays of all links, the vector will be sparse [27]. While the MR image shows sparsity after Fourier transformation, CS can be used for image reconstruction. In fact, the original paper of CS [10] was largely motivated by the reconstruction of MR imaging.

## 4. Sparse Signal Transmission Framework

In this section, we first present the background, which shows the characteristic of a lossy wireless link and the ubiquity of a sparse signal in sensor networks. Then, we reveal the process of sparse signal transmission and propose the problem formulation.

### 4.1. Background

#### 4.1.1. Lossy Wireless Link

The propagation of wireless signals with low-power radios is affected by several factors, such as space, time and and asymmetric connectivity [2], that contribute to the degradation of its quality. Consequently, wireless links in WSNs are often unpredictable and unreliable, especially lossy links in the transitional region, where the spatial characteristic of the wireless link presents three different communication regions, which are the connected region, the transitional region and the disconnected region [28].

To verify the spatial characteristic of the wireless link, we conduct extensive experiments by using a pair of STM32W108 chips [29] putting them in our teaching building corridor for link testing. The transmitting power for the sensor node is −10 dBm. The sender node transmits 256 packets to the receiver node in eachtest, with the packet length being 13 bytes. The experiment is conducted 10 times on each distance, and we take the typical packet reception rate (PRR) value to show the spatial characteristic.

**Figure 1:**
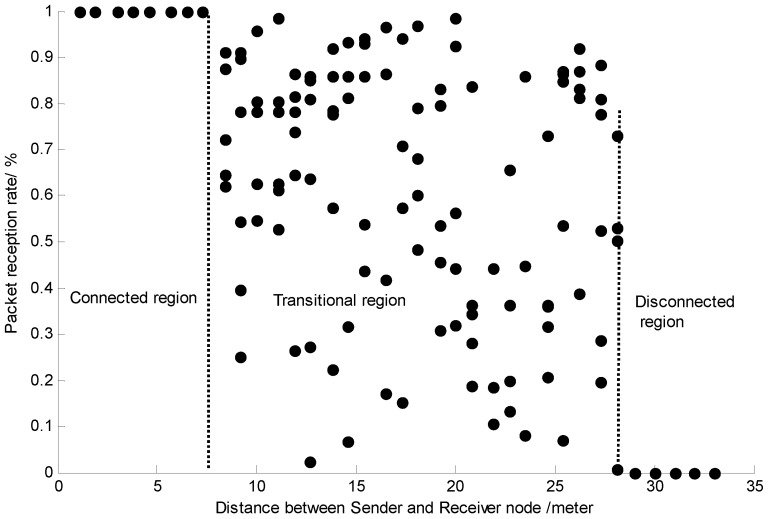
The spatial characteristic of the wireless link.

As shown in Figure 1, taking the PRR as the index to indicate the link’s quality, a higher PRR means better link quality. Figure 1 verifies the existing results for three different communication regions.

Traditionally, high quality links in the connected region are used to ensure communication reliability. However, a high quality link also implies shorter physical distance and, hence, denser sensor node deployment with higher transmission power and, hence, a shorter network lifespan. On the other hand, using an unstable lossy link in the transitional region invokes error-correcting measures, such as ARQ, to retransmit lost data packets. ARQ-based error-correcting measures will increase transmission times and latency. To this end, both approaches are expensive and imperfect solutions. In this section, we will introduce a novel use of the lossy link by using the compressive sensing technique.

#### 4.1.2. Ubiquity of Sparse Signals

It has been reported recently that many signals have sparsity in certain domains, such as temperature, light [15], image [10], acoustical signals [12], *etc*. Furthermore, [8] gives a relatively comprehensive summary of the signal’s sparsity with $CO_{2}$ emission readings [30], temperature [31] and the seismic signal [32].

**Figure 2:**
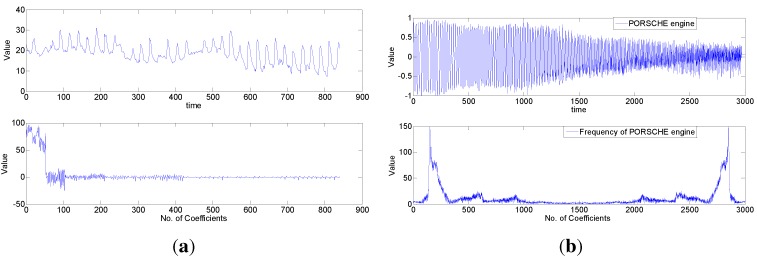
Signal sparsity in sensor networks. (**a**) Sparsity of $CO_{2}$ emission in the Port of Oakland; (**b**) sparsity of a car engine.

To verify the ubiquity of sparse signal, we take the $CO_{2}$ emission in the Port of Oakland and the acoustical signal of a Porsche car as examples to show their sparsity. Figure 2 shows their sparsity in the wavelet and frequency domains, respectively. Since signal sparsity is the fundamental condition for using CS, the ubiquity of a sparse signal in sensor networks enables one to apply compressive sensing to sparse signal transmission.

### 4.2. Process of Sparse Signal Transmission

According to the above discussion and former research, it only requires 20% of the whole data to recover the original signal (such as temperature and light [15]) with no more than 20% errors. While considering that the transitional region is formed mainly due to the path loss with small vibration caused by noise, with a 20% packet reception rate being settled to sufficiently reconstruct the original signal, a rough distance can be determined for stable use.

What is more, though the area of three regions in the spatial characteristic of the link varies with transmission environment, transmitting power *etc*., the transitional region occupies a large area compared to the connected region [28]. Stable use of a lossy link with our method can surely broaden the communication scope extensively.

**Figure 3:**
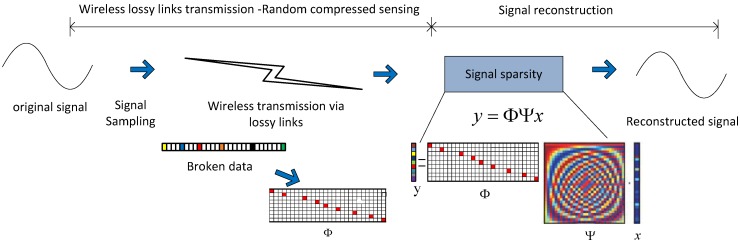
Process of reliable information transmission.

Next, we give a sketch of sparse signal transmission and then propose the problem formulation. The process of the CS-based reliable information transmission methods via lossy links is shown in Figure 3. It is composed of a lossy link transmission-random compressed sensing period and a signal reconstruction period. Instead of compressive sampling, we model the data packet loss as the measurement process. First of all, data obtained after signal sampling is directly transmitted through lossy wireless links. During wireless data transmission, there will be data loss. According to the CS theory, it is sufficient to capture the signal with a small number of compressive measurements. Thus, the sink node does not care which part of the information is successfully transmitted, as long as: (i) data loss caused by lossy links is random to some extent; (ii) there is a sufficient number of received packets to allow for the reconstruction of the information; and (iii) the original sequence of the received packets is known for the convenience of designing the projection matrix. Therefore, unlike the traditional methods, our proposed method can just disregard these lost packets. Then, based on the received broken data, the receiver node designs the projection matrix Φ through random compressive sensing (RCS) [33] as Equation (7) and gets the measurement data y=Φx. Finally, through transformation and orthogonal matching pursuit recovery algorithm, the original signal will be reconstructed from those received broken measurement data y.

### 4.3. Problem Formulation

The sensor node samples the signal and reports data to the sink node for signal recovery periodically. Supposing that the monitoring period includes Ntime slots. $x_{i}$ denotes the sensed data at time slot *i*, where i=1,2,....N.

Definition 1 Original Data Vector . This is the mathematical presentation of the original signal after signal sampling, which is defined as $x=(x_{1},x_{2}...x_{N})$. **x** represents every data sampling in N time slots without missing data elements.

Definition 2 Lossy Transmission Matrix. This is also the projection matrix, whose matrix dimensions are M×N, recording the data loss process caused by a lossy link. *M* is the received data number. The matrix has only a single entry of one in each row, and the others zero. The value in projection matrix **Φ** can be chosen through the following formula:

(7) $$\Phi \left(i,j\right)=\left\{\begin{array}{c} 1\;\;if\;j=J(i)\leq N\\ 0\;\;\;\;\;\;\;\;\;otherwise \end{array}\right.$$

where *i* is the row number of **Φ** and also the received data packets’ sequence number. J(i) is the received data’s original sequence number in *f*.

The effective projection matrix corresponds to a partial Fourier transform projection matrix, which has been shown to meet the restricted isometry property (RIP) and, hence, promises the reconstruction of the sensor signal at the fusion center with a high probability. The feasibility of this approach has been reported earlier in [12,33,34].

**Figure 4:**
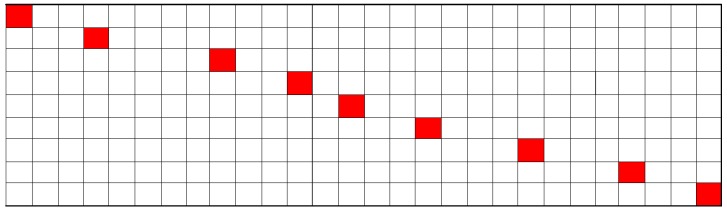
An example of the projection matrix construction.

Suppose a signal consisting of 28 sampled data is transmitted through a wireless medium and only nine sampled data are successfully received in the FC, with their sampled sequence number being 1, 4, 9, 12, 14, 17, 21, 25, 28. Then, the corresponding projection matrix is shown in Figure 4, where the blank grid denotes corresponding elements being zero and red being one.

Definition 3 Measurement Vector. This is a N×1 vector. It is the combination of data packets received after lossy wireless transmissions. Due to the data loss, its elements are either $x_{i}$ gathered by the sensor node or zero. It can be denoted as the production of Φ and *x*,

(8) y=Φx

which conforms to the standard compressive sensing expression.

Definition 4 Reconstructed Vector. This is generated by reconstruction algorithms and denoted as $\hat{x}=\left({\hat{x}}_{1},{\hat{x}}_{2}...{\hat{x}}_{N}\right)$.

Problem: Original Signal/Information Reconstruction . Given the measurement vector *y*, this problem is to find an optimal reconstructed vector $\hat{x}$ that approximates the original data vector *x* as closely as possible. *i.e.*,

(9) $$\begin{array}{c} \mathrm{Objective}\;\;\;\;\;\min||x-\hat{x}|{|}_{2}\\ \mathrm{Subject}\;\;\mathrm{to}\;\;\;\;\;\;y \end{array}$$

where $\left||.\right|{|}_{2}$ is the $l_{2}$ norm used to measure the error between vector *x* and $\hat{x}$.

In the above problem, the objective is to minimize the absolute error. In order to measure the reconstruction error, we further define the following metric.

Definition 5 Reconstruction Error. We define the parameter of the reconstruction error *ε* as follows:

(10) $$\varepsilon =\frac{\sqrt{{\sum_{i}\left(x_{i}-{\hat{x}}_{i}\right)}^{2}}}{\sqrt{{\sum_{i}x_{i}}^{2}}}$$

Reconstruction error reflects the similarity degree of the reconstructed signal and the original one. The smaller the reconstruction error is, the higher the data recovery accuracy of the reconstruction methods will be.

Definition 6 Information Acquisition Rate (IAR). Consider that traditional link quality estimation usually adopts packet reception rate, which is based on data acquisition. Aiming to measure the information transmission quality of a certain link, we define a new link performance metric called the information acquisition rate (IAR) as follows, where thresholddenotes the application’s need for signal recovery error. For example, in the practical application of structural health monitoring (SHM), the reconstruction error should be less than 30%, so as to satisfy the engineering requirement [35], which means threshold=0.3.

(11) $$IAR=\left\{\begin{array}{cc} 1 & 0<\varepsilon <threshold\\ 0 & \varepsilon >threshold \end{array}\right.$$

## 5. Packet Length Control for Sparse Signal Transmission

Considering the unit of wireless communication is packet and packet length control in a traditional communication system has brought remarkable performance promotion, we analyze the packet length control for sparse signal transmission in this section. Supposing the sampling precision is 8 bits, therefore the number of samples in one packet is the payload length.

### 5.1. Packet Length Effect on Mutual Coherence

**Figure 5:**
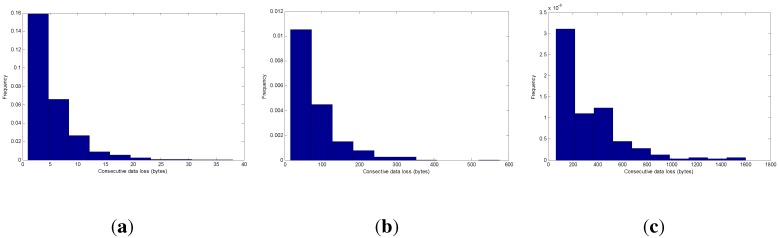
Frequency histogram of consecutive data loss. (**a**) Consecutive loss with the payload length being one byte; (**b**) consecutive loss with the payload length being 16 bytes; (**c**) consecutive loss with payload length being 64 bytes.

A trade-off exists between the desire to reduce overhead by making packets large and the need to reduce packet error rates in the noisy channel by using small packet length. As shown in Figure 5, supposing a signal consisting of 64 KB of sampled data is transmitted through a wireless medium, with the PRR being 20%, when the payload length increases, once a packet has failed to transmit, more consecutive data will be lost, leading to bursty data loss. Traditionally, bursty data loss is difficult to recover from using interpolation-based methods [15]. Next, we will show the packet length effect on sparse signal transmission.

We first introduce mutual coherence as the metric to measure signal transmission quality and then reveal the relationship between packet length and signal recovery performance. The mutual coherence, denoted as μ(A),

(12) $$\mu \left(A\right)=\max_{i\neq j,1\leq i,j\leq N}\left\{\frac{|{A_{i}}^{T}A_{j}|}{\left|\right|A_{i}\left||.|\right|A_{j}\left|\right|}\right\}$$

represents the worst case coherence between any two columns (atoms) of equivalent matrix *A* and is one of the most fundamental quantities associated with CS theory. Any *K*-sparse signal *x* can be exactly recovered from the observation/measurement y=Ax as long as:

(13) $$K<\frac{1}{2}\left[1+\frac{1}{\mu (A)}\right]$$

Another suitable way to describe μ(A), which is also our method in this paper, is to compute the Gram matrix $G={\tilde{A}}^{T}\tilde{A}$, where $\tilde{A}$ is column-normalized version of *A* [36]. Then:

(14) $$\mu \left(A\right)=\max_{i\neq j,1\leq i,j\leq N}\left|g_{ij}\right|$$

**Figure 6:**
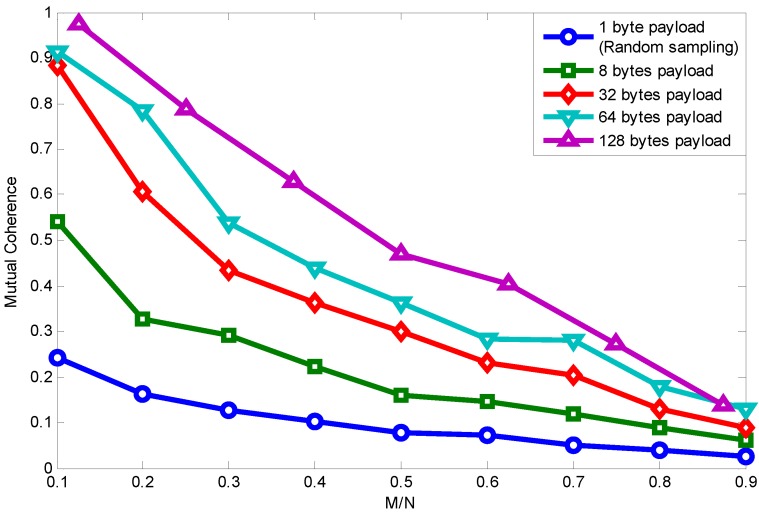
Packet length effect on relationship between $\frac{M}{N}$ and mutual coherence using random compressive sensing (RCS) projection.

Figure 6 shows the relationship between mutual coherence and packet length with the RCS projection matrix. For convenience, we make the signal dimension *N* be divisible by payload length. Because the Fourier basis requires *N* to be a power of two, the payload length is chosen to be 1, 8, 32, 64, 128. Though it is not practical using a payload length of 128 bytes, since the maximum packet length in the IEEE 802.15.4 standard is 127 bytes, we only draw it as a reference here by using a 127-byte length to approximate the 128-byte length.

Specifically, when the payload length is one byte, it is similar to random sampling [33]. We can see that when the measurement vector is fixed, mutual coherence increases with the growing length of the packet. Furthermore, as the measurement vector number increases, the coherence difference caused by packet length is weakened.

However, this is just a clue to the relationship between packet length and signal recovery performance; signal recovery performance is also correlated with other factors, such as signal sparsity. According to [8], the sparsity of typical signals in sensor networks, such as temperature and light, is between 5 and 10. Based on this, we show the relationship between mutual coherence and reconstruction error as follows.

**Figure 7:**
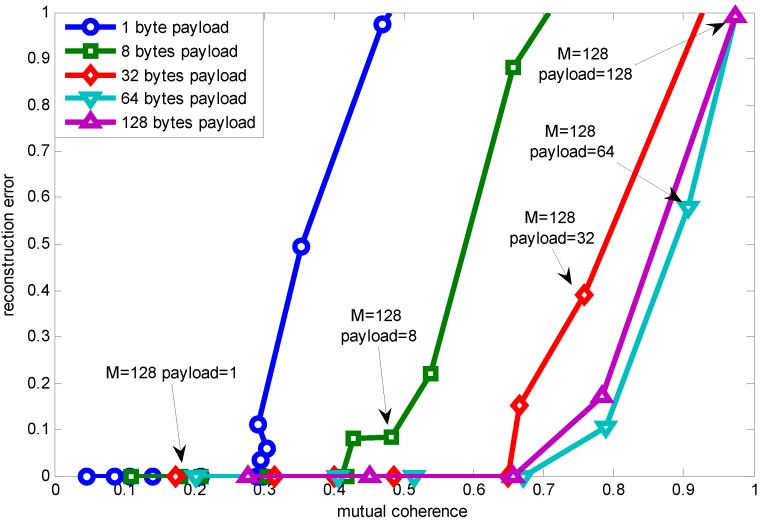
Relationship between mutual coherence and reconstruction error using RCS projection with K = 5.

Figure 7 shows a noteworthy phenomenon, that as packet length increases, the mutual coherence guaranteeing accurate reconstruction (error being zero) is increasing, as well (except when the payload length is 128 bytes, which is due to its low resolution, making us not find an appropriate value). This figure is drawn through changing the measurement number when different payload lengths are adopted. The reason for the packet length effect is when the measurement number is fixed, a shorter packet length achieves lower mutual coherence. While reconstruction accuracy is correlated with measurement number, as shown in Equation (3), when the measurement number meets the need for recovery, a longer packet length has larger coherence compared to shorter packet length.

**Figure 8:**
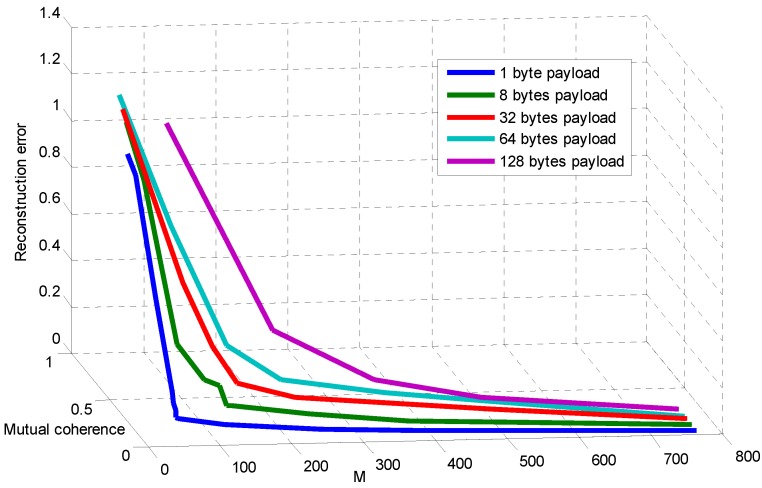
Relationship between measurement number, mutual coherence and reconstruction error.

Further, Figure 8 shows the relationship between measurement number, mutual coherence and reconstruction error when different payload lengths are adopted. Generally, the measurement number has a direct effect on mutual coherence, which influences the reconstruction error. However, packet loss with different packet lengths also affects the mutual coherence, as shown in Figure 6. Figure 8 presents the combined effect of packet length and measurement number on mutual coherence (further on recovery error). Because a larger packet length decreases the mutual coherence, the measurement number guaranteeing the recovery error increases compared to the shorter packet length.

### 5.2. Relationship between Communication Parameter and Mutual Coherence

Packet length does not directly act on mutual coherence/signal transmission quality. In fact, its effect combined with the bit error rate (BER) can determine the packet reception rate (PRR) and then influence the signal transmission quality.

BER is a fundamental parameter that can be determined by transmitting power, modulation scheme and transmission distance. In practical applications, transmission distance is fixed once sensor nodes are deployed, as well as the modulation scheme with specific hardware chips, for example chips that offer the IEEE 802.15.4 standard communication adopt the offset-quadrature phase shift keying (O-QPSK) modulation scheme. Without considering transmitting power control in this paper, BER is almost settled. What is more, BER is the key parameter that determines the quality of the wireless channel.

This part first presents the relationship between BER, packet length and PRR, then shows how these communication parameters affect signal transmission quality.

#### 5.2.1. Combined Effects of BER and Packet Length on PRR

Given bit error rate $P_{e}$, the packet reception rate $P_{prr}$ is obtained as follows:

(15) $$P_{prr}={\left(1-P_{e}\right)}^{L}$$

where $L=L_{overhead}+L_{payload}$ denotes the whole packet length including overhead. $P_{e}$ depends on the modulation scheme. For binary phase shift keying (BPSK) or quadrature phase shift keying (QPSK) adopted by the IEEE 802.15.4 standard at 2.4 GHz, the bit error rate is the same and equals:

(16) $$P_{e}=Q\left(\sqrt{\frac{2E_{b}}{N_{0}}}\right)=Q\left(\sqrt{2\beta }\right)$$

where *β* is the $\frac{E_{b}}{N_{0}}$ ration. The *Q*-function is defined as Q(x)=∫x∞12πexp(-x2-x222)dx=12[1-erf(x2)]. Hence, the packet reception rate $P_{prr}$ is defined as:

(17) $$P_{prr}={\left(1-Q\left(\sqrt{2\beta }\right)\right)}^{L}$$

We now conjecture that the successfully-received packet number by the FC denoted as *M* has a binomial distribution with parameter $n=\frac{N}{L_{payload}}$ and probability $P_{prr}$, *i.e*.,

(18) $$P\left(M^{\prime }=m\right)=B\left(n,P_{prr}\right)=C_{n}^{m}{P_{prr}}^{m}{\left(1-P_{prr}\right)}^{n-m}$$

where *n* is the total transmitted packet number. The measurement number (*M*) is:

(19) $$M=n\times P_{prr}\times L$$

where *N* denotes the number of sampling data. In this paper, we use N=1024. In this case, given a deterministic channel condition and packet length, we can acquire the quality of signal/information transmission.

#### 5.2.2. Threshold of PRR for Reliable Signal Reconstruction

Therefore, given the required packet reception rate $p_{threshold}$, appropriate parameter selection can achieve accurate sparse signal transmission as long as the following condition is satisfied:

(20) $$P_{prr}={\left(1-Q\left(\sqrt{2\beta }\right)\right)}^{L}\geq p_{threshold}$$

where:

(21) $$p_{threshold}=\frac{M}{N}=\frac{cK\log\left(N/K\right)}{N}$$

with *K* being the signal sparsity and *c* a constant.

**Figure 9:**
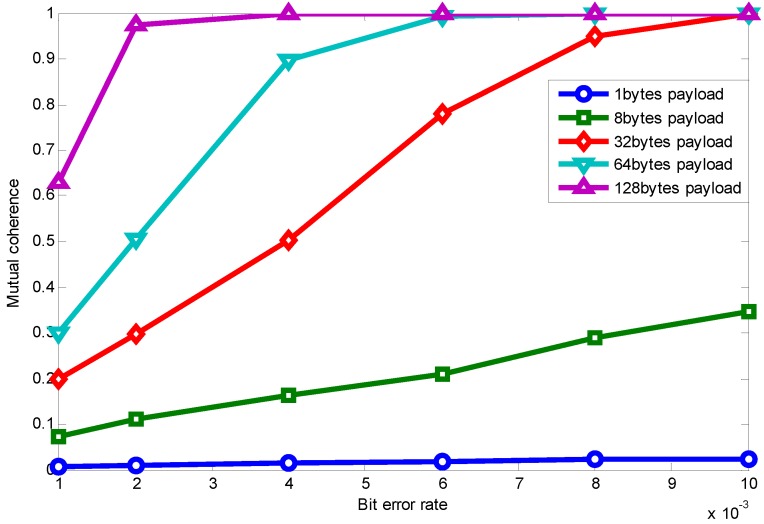
Packet length effect on the relationship between BER and mutual coherence using RCS projection.

Figure 9 shows the packet length effect on the relationship between BERand mutual coherence using RCS projection, which shows that mutual coherence increases when the channel condition becomes worse and the packet length becomes longer. Considering specific applications (specific signal sparsity) and the relationship between mutual coherence and recovery error, as shown in Figure 7, the application’s requirements can be met through appropriate parameter selection.

### 5.3. Performance Improvement with Data Interleaving

As mentioned in the above part, a longer packet length has a bad effect on signal recovery. In this part, we use the traditional communication technique to improve it. We first introduce the interleaving technique and then show its effect on sparse signal transmission by analyzing mutual coherence, the relationship between mutual coherence and recovery error and the relationship between mutual coherence and bit error rate (BER). At last, considering that interleaving length is limited by memory size and the application’s timeliness, the relationship between mutual coherence and interleaving length is investigated.

#### 5.3.1. An Easy-to-Implement Method: Interleaving

Traditionally, when data loss exists, ARQ is always adopted to retransmit lost packets. However, ARQ does not function well when severe packet collision or communication noise exists with introducing extra energy consumption and delay. There is something else we can do that provides better protection against burst errors: we can transmit data bits in a different order than the order in which the data bits were originally transmitted. Doing so is called interleaving [37]. Adopting data interleaving before data transmission as a feedforward way surely can make the data loss random, thereby improving the sparse signal transmission performance in bursty data loss condition. This approach is based on mixing up or interleaving the order of the sampling sequence before transmission and unmixing or de-interleaving it on reception. That way, if a packet (or burst of packets) is lost, the loss will be spread out over time by the mixing. It will not result in a single, large gap during signal transmission.

#### 5.3.2. Interleaving Effect on Sparse Signal Transmission

In this part, we present the interleaving effect on sparse signal transmission by analyzing mutual coherence, the relationship between mutual coherence and recovery error and the relationship between mutual coherence and BER.

**Figure 10:**
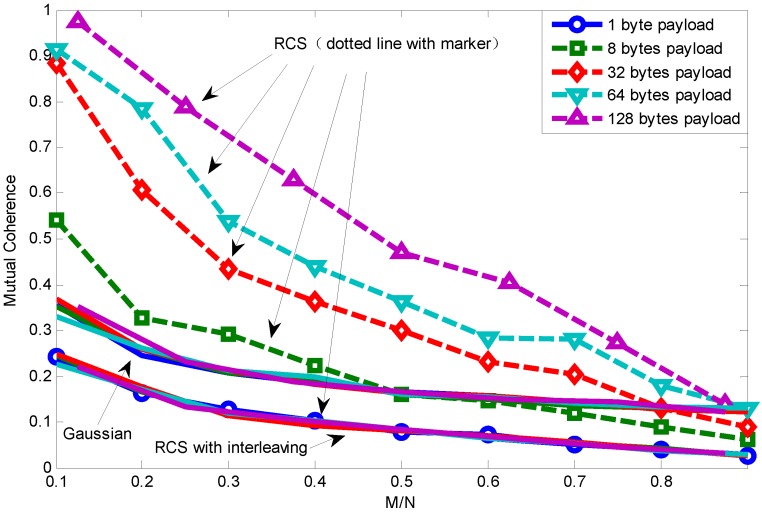
Relationship between mutual coherence and packet length using RCS projection with interleaving.

Figure 10 shows the relationship between mutual coherence and packet length using RCS projection with interleaving. We can see that data interleaving actually lower the mutual coherence of equivalent matrix *A*. This is because data interleaving makes data loss more random, which helps to make projection matrix and basis matrix more incoherent. It is also noteworthy that the performance of using RCS with interleaving as the projection matrix is even better than using the random Gaussian projection matrix.

**Figure 11:**
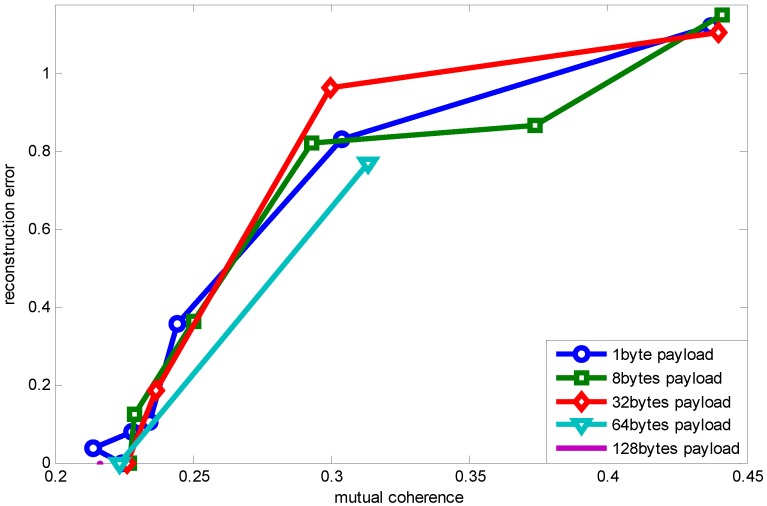
Relationship between mutual coherence and reconstruction error using RCS projection with interleaving.

Next, Figure 11 shows the packet length effect on the relationship between mutual coherence and reconstruction error using RCS projection with interleaving. We can see that packet length has little effect on the relationship between mutual coherence and reconstruction error. This is because interleaving makes data loss random whichever packet length it is, therefore eliminating the packet length effect. Since when signal sparsity is five, one packet with the payload length being 128 bytes can recover the original signal, the curve corresponding to a 128-byte payload is a point.

**Figure 12:**
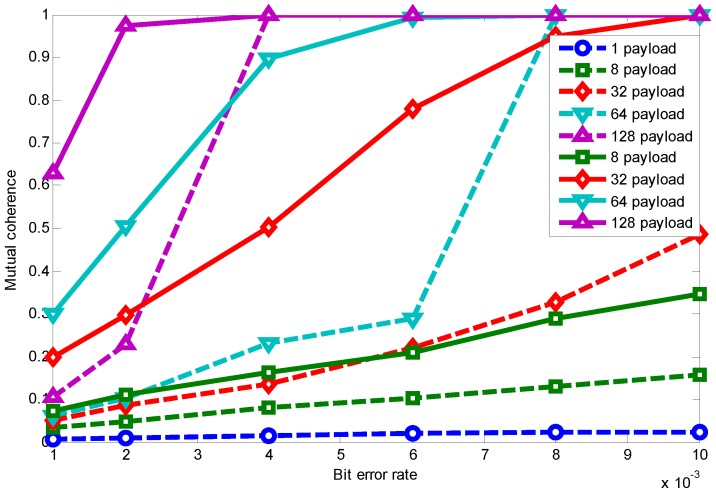
Packet length effect on relationship between BER and mutual coherence using RCS projection with interleaving.

Figure 12 shows the packet length effect on the relationship between BER and mutual coherence. The dotted line is drawn using RCS projection with interleaving, while the solid line is obtained by using only RCS projection. This shows that mutual coherence increases when the channel condition becomes worse and the packet length becomes longer. However, as long as there is a received packet, interleaving can lower the mutual coherence under the same channel condition and packet length, therefore promoting the sparse signal transmission performance. Considering the specific application (specific signal sparsity) and relationship between mutual coherence and recovery error, as shown in Figure 11, appropriate parameter selection can be made to meet the application’s requirements.

#### 5.3.3. Interleaving Length

**Figure 13:**
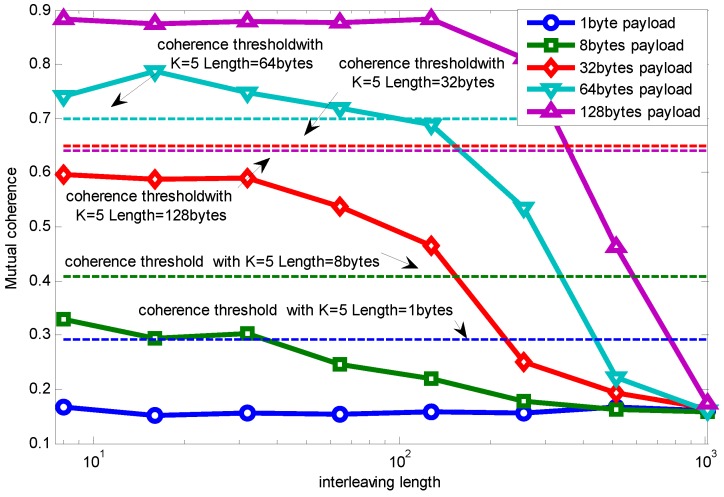
Relationship between mutual coherence and interleaving length.

Note that performance improvement is correlated with interleaving length, which is limited by memory size and the application’s timeliness. Figure 13 further shows the relationship between mutual coherence and interleaving length when $\frac{M}{N}=0.2$.

The dashed line represents the mutual coherence guaranteeing accurate signal recovery (the error being zero) when using RCS as the projection matrix with packet lengths equal to 1, 8, 32, 64, 128 bytes. A larger interleaving length makes the data loss more random, therefore making the mutual coherence smaller. Longer packets lead to longer bursty loss, therefore needing larger interleaving length. However, larger interleaving length induces larger transmission delay and needs more memory space. A trade-off must be made between the application’s requirements (signal recovery accuracy, latency, *etc.*) and interleaving length, which will be our further work. In the following part of this paper, we leave this problem by considering the interleaving length equal to *N*.

## 6. Performance Evaluation

In this section, we take the sound signal consisting of 30 different frequencies (K = 30, much larger than the temperature’s sparsity [8]) as the original signal needed to be transmitted. The acoustical signal is sparse in the Fourier domain, which is fundamental for using compressed sensing. We use ns3 to simulate the random data packet loss procedure, where wireless links with different qualities are obtained through changing the distance between the sender node and the receiver node. The default backoff exponent of the IEEE 802.15.4 standard is adopted, and Channel 11 of the standard is used considering the difference between different channels.

The main parameters used in NS-3 simulation are listed in Table 2. Our CS-based methods do not use the data retransmission scheme, even if data loss happens during wireless transmission, while the traditional ARQ interpolation methods adopt a maximum of three-times the transmission scheme. There are four typical interpolation methods adopted in ARQ interpolation methods, which are nearest neighbor (NN), cubic polynomial interpolation, spline interpolation and linear interpolation. Upon data packet reception, we use the orthogonal matching pursuit algorithm or L1-Magic to reconstruct the original signal, comparing to the traditional interpolation method.

### 6.1. Performance Comparison on Signal Reconstruction

In this part, to eliminate the effect of packet length on sparse signal transmission, we fix the payload length to one byte and model the data loss as random compressive sampling. Then, the original signal can be reconstructed by using the received broken data. The original packets’ number is N=1024.

**Table 2:** Parameter settings in NS-3 simulation.

Tx Power	0 dB
Channel number	11
Simulation time	4 s
Max frame retries	0 or 2
Overhead length	10 bytes
Minimum packet length	11 bytes
Maximum packet length	127 bytes
Minimum distance between sender and receiver	98 m
Maximum distance between sender and receiver	128 m

With compressive sensing, it only needs 20% of the data to recover the real signal, such as temperature and light [15]. Furthermore, conducting DOA estimation using 20% data is also sufficient [38]. To verify the effectiveness of our CS-based method, we first conduct simulations over a link in the transitional region, in which the packet reception rates (single data transmission) are 20%.

**Figure 14:**
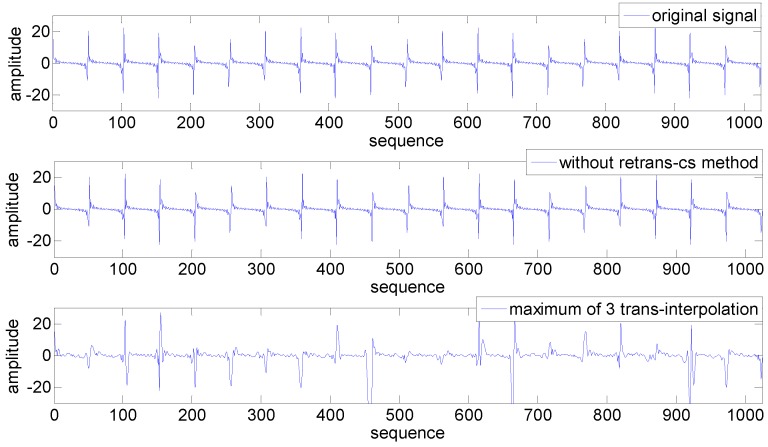
Reconstruction comparison.

The reconstruction comparison is shown in Figure 14. The interpolation method in this test is spline, which is thought to be more efficient than other methods. As we can see, when the PRR is 20%, data loss is quite severe. Even with a maximum of three transmission schemes, the number of received packets still cannot satisfy the interpolation’s need to reconstruct the signal accurately. The traditional interpolation method can only recover a few parts of the signal with received broken data, leading to large recovery error, which means adopting the ARQ interpolation-related technique in harsh environments is not very useful, but has extra energy waste and transmission latency; while due to the sparsity of the original signal and the appropriate selection of the projection matrix, a small number of compressive measurements can capture the signal with high probability and high precision. By using the CS reconstruction algorithms, our CS-based method can recover the original signal with a much smaller reconstruction error.

**Figure 15:**
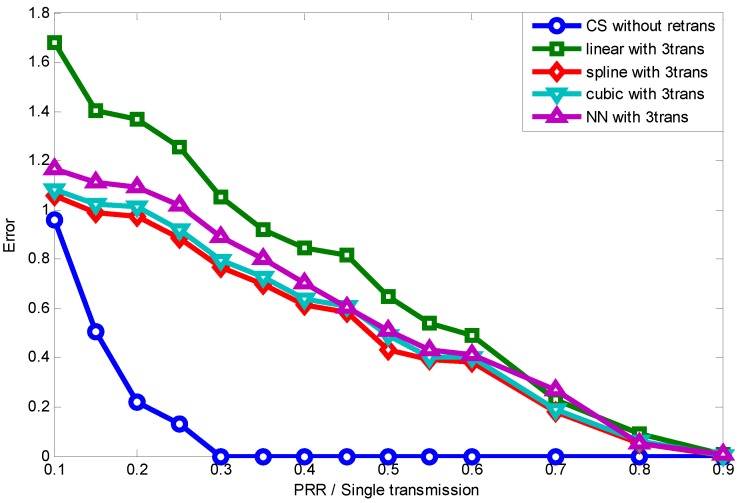
Reconstruction error comparison.

Figure 15 further presents the reconstruction error comparison between CS and interpolation methods under different lossy links (different PRR). Figure 15 shows that all four ARQ interpolation methods perform badly when the PRR is below 0.6, even with three-times the data transmission. Just as described previously, interpolation methods are suitable only for a few data loss condition; while thanks to the sparsity of the original signal and the appropriate selection of projection matrix, a small number of compressive measurements can capture the signal with high probability and high precision. As we can conclude from Figure 15, spline interpolation performs the best when the PRR is below 0.6. In the following simulation, we take spline interpolation as the comparison with our CS-based method.

To better show how much energy waste and transmission latency can be reduced by using our CS-based method for information transmission in lossy links, we take the recovery error of the CS-based method as a reference to show how many transmissions are needed by the ARQ interpolation method to achieve the same error. Given the PRR per single transmission, the PRR with Ntrans times transmission is theoretically:

(22) $$P_{prr}^{Ntrans}=1-{\left(1-P_{prr}\right)}^{Ntrans}$$

Therefore, given the packet needed $M_{need}$ for interpolation to achieve the same recovery accuracy, the corresponding $P_{prr}^{Ntrans}$ can be obtained as below:

(23) $$P_{prr}^{Ntrans}=\frac{M_{need}}{N}$$

Then, once the single transmission PRR $P_{prr}$ is known, the transmission times needed are represented by:

(24) $$Ntrans=\frac{\log(1-P_{prr}^{Ntrans})}{\log(1-P_{prr})}$$

**Figure 16:**
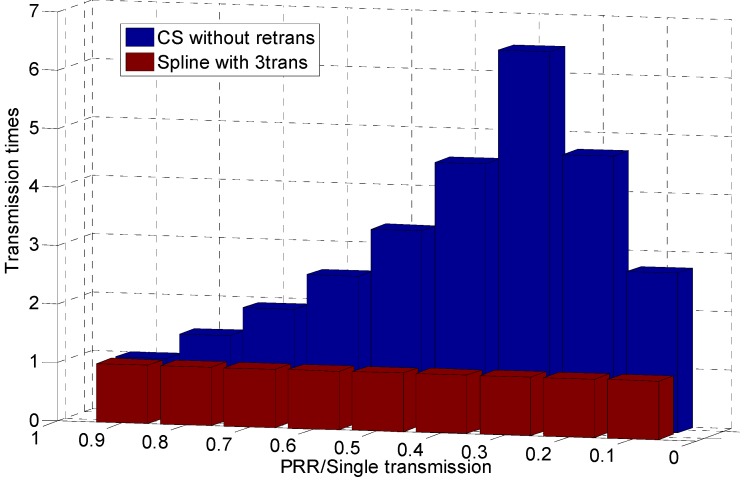
Required transmission times achieving compressive sensing’s (CS) error.

Figure 16 shows that when the link quality is extremely low, both the CS method and ARQ interpolation method cannot recover the original signal accurately, since many data packets have not been successfully received. As the quality of links improves, the CS method can recover the signal when the packets received exceed the threshold, as Equation (3) shows, while the ARQ interpolation method’s performance is linearly promoted with the PRRs increasing. When the PRR of single data transmission is 0.3, ARQ interpolation need 6.5-times transmission to achieve the same performance as CS-based method. Even when the PRR is 0.8, nearly 1.5-times transmissions are still needed to achieve the same reconstruction accuracy as our method. All in all, the CS-based method can enable almost all of the lossy links in the transitional region to provide high quality information transmission and also significantly reduces the energy cost and latency.

**Figure 17:**
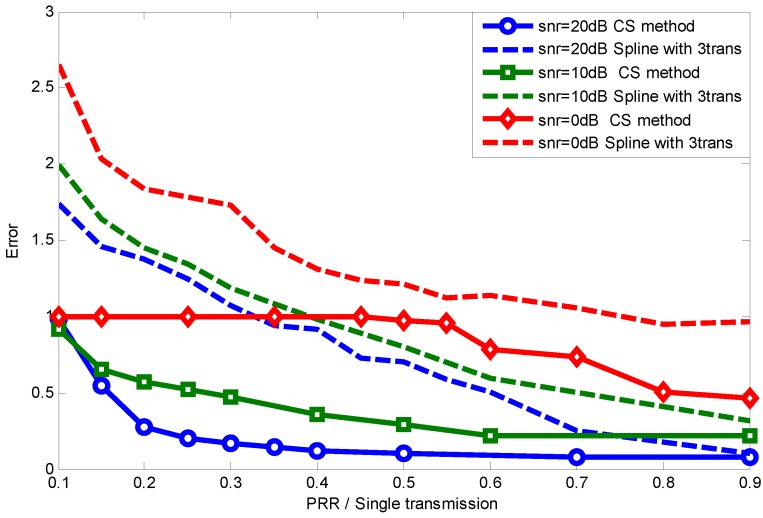
Signal reconstruction error comparison with noise corruption.

Further, considering that noise usually corrupts the data sampling in a real scenario, the robustness comparison between our CS-based method and ARQ interpolation is of great significance. Figure 17 shows the signal reconstruction error comparison with noise corruption. We can see that noise increases the recovery error, and the CS method outperforms the spline-multiple transmission methods generally. This is because CS reconstruction algorithms can adopt the noise threshold to improve the recovery performance, while the traditional interpolation method does not have this function.

### 6.2. Packet Length Effect on Sparse Signal Transmission

Based on the relationship between packet length and mutual coherence, we present the performance evaluation of signal transmission efficiency with varying packet length in this part. Compared to using successfully transmitted data times as the efficiency criterion, we take the IAR at the fusion center (FC) as the criterion to measure the efficiency of signal/information transmission.

Specifically, considering that the reconstruction error should be less than 30%, so as to satisfy the engineering requirement in the practical application of structural health monitoring (SHM) [35], we set threshold=0.3 in this part. Furthermore, for a high quality requirement application, we set threshold=0.001 compared to threshold=0.3 in SHM applications.

#### 6.2.1. Performance under Varying BER

BER is a fundamental parameter that can be determined by transmitting power, modulation scheme and transmission distance. In practical applications, transmission distance is fixed once sensor nodes are deployed, as well as the modulation scheme with chosen hardware chips, for example chips that offer the IEEE 802.15.4 standard communication adopt the O-QPSK modulation scheme. Without considering transmitting power control in this paper, BER is almost settled. What is more, BER is the key parameter that determines the quality of the wireless channel. Therefore, next, we consider the sparse signal transmission performance under varying BER.

If the BER is too high, for example BER = 0.1, the packet length is 10 bytes, even without the payload. According to Equation (15), PRR = $2.1847\times 10^{-4}$. With such a small measurement number, CS cannot recover the sparse signal. Both the data and signal transmission efficiency are zero.

**Figure 18:**
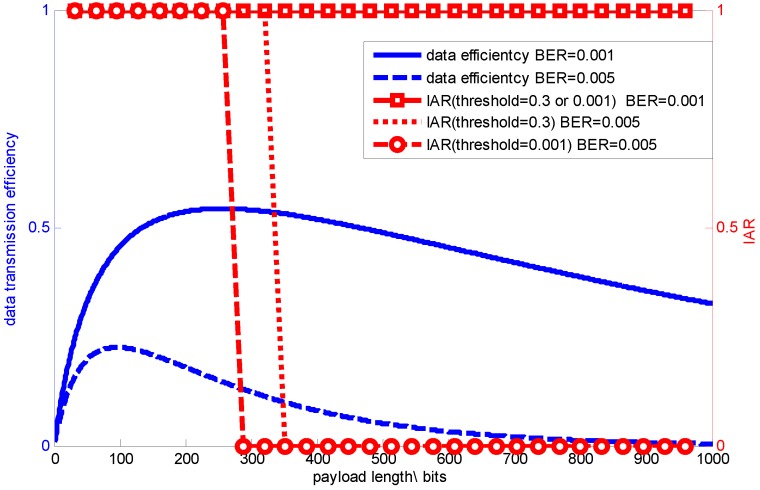
Comparison between data and signal transmission efficiency.

As BER increases, for example BER=0.005, using a signal with sparsity K=5, comparison between data and signal transmission efficiency is shown in Figure 18.

Note that, when the payload length equals 10 bytes, we have the best data transmission efficiency as being around 0.24. However, for a signal with sparsity K=5, it can achieve almost the total signal transmission efficiency with recovery error $\varepsilon =3.6191\times 10^{-15}$, since it meets the need for measurement number used for signal reconstruction. Additionally, in this case, the optimal packet length for this sparse signal transmission is around 35 bytes, other than 10 bytes.

What is more, when BER decreases, for example BER=0.001, even using the longest payload length specified in IEEE 802.15.4, it can still meet the measurement number threshold, which means that signal transmission efficiency is always one, even when the payload length varies, as shown in Figure 18. Based on the above discussion, the optimal packet length is determined by both the specific application requirements and channel condition.

**Figure 19:**
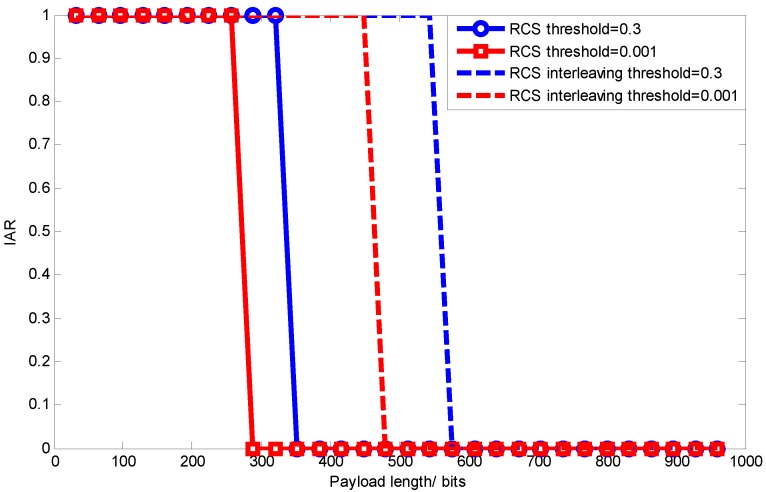
Signal transmission efficiency comparison after data interleaving.

Figure 19 shows the signal transmission efficiency comparison between using RCS and RCS with data interleaving when BER = 0.005. We can see that signal transmission efficiency varies as the application threshold for signal recovery changes. When the threshold is larger, a larger packet length can be chosen under the same channel condition (same BER). While data interleaving mitigates the bursty loss, RCS with data interleaving also allows one to use a larger packet length.

#### 6.2.2. Performance under Varying Signal Sparsity (Various Applications)

**Figure 20:**
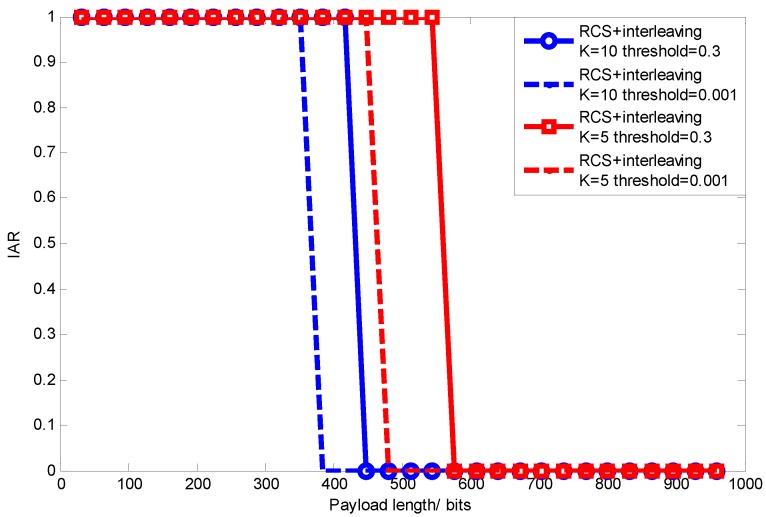
Signal transmission efficiency with varying sparsity.

Since the measurement number threshold for signal reconstruction is correlated with sparsity, which is determined by the specific application, thus varying sparsity changes the signal/information transmission efficiency. Considering the sparsity of many signals, such as seismic wave, temperature, a density of $CO_{2}$ being about 5 to 10 [8], we use signals with K=5 and K=10 to show the signal’s impact on signal transmission efficiency. Figure 20 shows the relationship between signal transmission efficiency and packet length under various signal sparsities when BER=0.005 and BER=0.01. Apparently, the signal transmission efficiency is getting worse with the increasing signal sparsity, which makes a smaller optimal packet length. This is due to the reason that larger signal sparsity needs a greater measurement number to accurately reconstruct the sparse signal.

## 7. Experimental Verification

**Figure 21:**
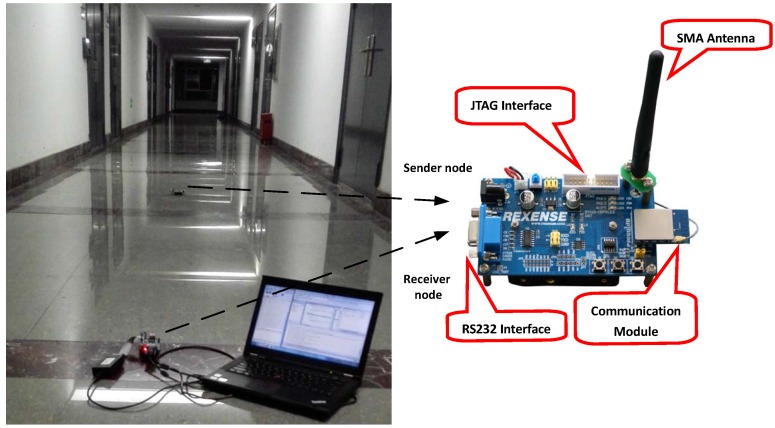
Spatial characteristic of the wireless link.

In this section, we first use a pair of STM32W108 chips in our building corridor to conduct the lossy link transmission experiment, as shown in Figure 21, to obtain the lossy packet pattern, and this loss pattern is further utilized to show the packet length effect on mutual coherence and recovery error for sparse signal transmission. A real Porsche car sound, as shown in Figure 2b, is sampled, and its data then can be transmitted to the receiver node. There are more than 160 coefficients that are larger than five after Fourier transformation. Various packet reception rates are obtained through changing the distance between the sender and receiver nodes. The transmitting power is −10 dBm for both the sender and receiver nodes, and there is no retransmission during this experiment. This section aims to further verify sparse signal transmission performance, the packet length effect on mutual coherence and the recovery error presented in the simulation results of the last section. We first show that sparse signal transmission using compressive sensing outperforms traditional methods. The packet length effect is present to show that a larger packet length has an adverse impact, which increases mutual coherence and, thus, increases recovery error with the same M/N. Then, with the knowledge that correctly-received packets correspond to a deterministic projection matrix, which shows inefficiency when using a large packet length, we further verify the interleaving improvement on mutual coherence and recovery error, respectively.

### 7.1. Sparse Signal Transmission Performance

**Figure 22:**
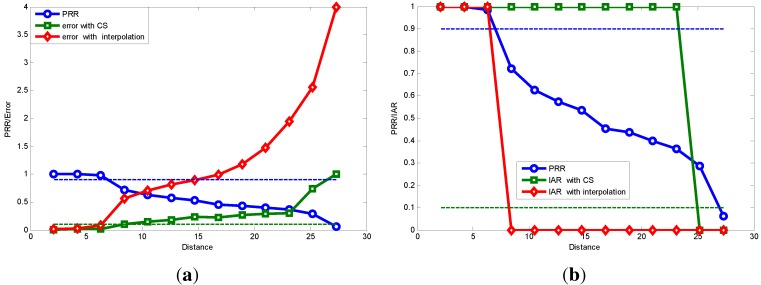
Sparse signal transmission performance. (**a**) Packet reception rate (PRR) and recovery error comparison with varying transmitting distance; (**b**) PRR and information acquisition rate (IAR) comparison with varying transmitting distance.

Figure 22a shows the packet reception rate and recovery error comparison with varying transmitting distance. The distance area between the blue and green dotted link is the transitional region in the spatial characteristic of the lossy link; these two dotted lines respectively represent the 90% and 10% packet reception rates [28].We can see in the transitional region that using compressive sensing-based sparse signal transmission outperforms the transitional interpolation methods. This is because the compressive sensing method can recover the original sparse signal using only part of its sampled data, and the compressive sensing recovery algorithm is resistant to the noise in the signal.

Figure 22b further shows that our methods expand the feasible communication range taking SMH application’s requirement as an example [35]. The work in [35] states that the reconstruction error should be less than 30%, so as to satisfy the engineering requirement in practical the application of SHM. Therefore, next, we denote the recovery error threshold to be 30%, which means that the IAR equals one if the recovery error is less than 30%, and the IAR equals zero otherwise, as specified in Equation (11). In Figure 22b, we can see that when the transmitting power is −10 dBm, the range of the connected region is around 7 m and the range of transitional region is about 19 m, which is from 8 m to 27 m. Using traditional interpolation methods, one can only use the connected region for communication. While using compressive sensing-based methods, one can expand the feasible communication range from 7 m (connected region) to 23 m. The feasible communication range is promoted by almost 230%. This is only the result when the transmitted power is −10 dBm, and we know that the larger the transmitting power is, the larger the relative range is between the transitional region and the connected region [28]. We can conclude that when the transmitting power is 0 dBm, which is practical, our method’s superiority will become even more prominent.

**Figure 23:**
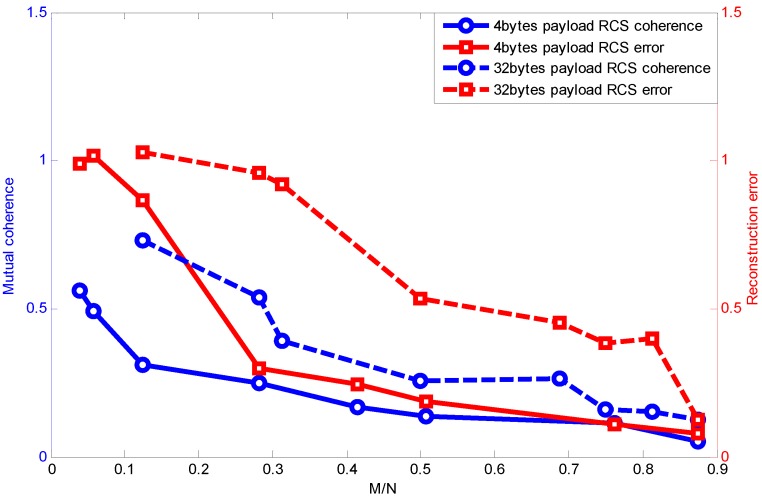
Mutual coherence and recovery error comparison with different packet lengths.

### 7.2. Packet Length Effect Verification

Figure 23 shows the mutual coherence and recovery error comparison with the packet length being a four-byte payload and a 32-byte payload, respectively. Just as the simulation results presented, when M/N is the same, a larger packet length yields larger mutual coherence, and as the M/N increases, the coherence difference caused by the packet length decreases. Furthermore, when M/N is the same, a shorter packet length generally can achieve a lower recovery error, and once its measurement number exceeds the threshold required for accurate recovery (Equation (3)), its recovery error diminishes drastically. Note that the value of the error in this part may be different from the recovery error in the simulation results, which is because the signal used in the experiment has a much larger sparsity. Another reason is that the signal has been corrupted with noise in the data sampling process. However, the trend is the same, which is consistent with the simulation results.

### 7.3. Interleaving Improvement Verification

In this part, we further verify the interleaving improvement as presented by the simulation results earlier.

**Figure 24:**
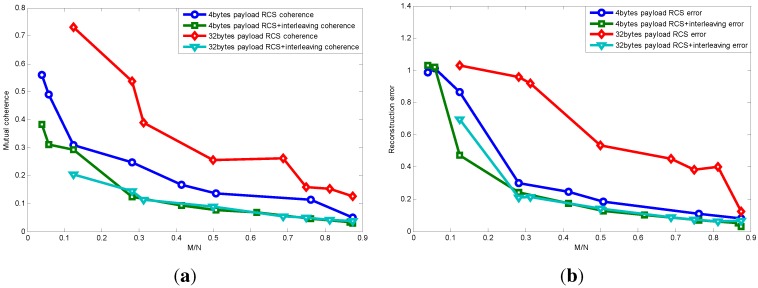
Interleaving improvement with different packet lengths. (**a**) Interleaving improvement on mutual coherence with different packet lengths; (**b**) interleaving improvement on recovery error with different packet lengths.

Figure 24a shows the interleaving improvement on mutual coherence with different packet lengths. We can see that using only RCS as the projection matrix, when M/N is the same, a larger packet length yields larger mutual coherence, and as the M/N increases, the coherence difference caused by packet length decreases. While using RCS with the interleaving technique, their mutual coherence is almost the same with identical M/N. When considering that coherence increases with a larger packet length using RCS projection, we attribute the cause to the larger packet length, making data loss bursty, which is bad for signal recovery; while data interleaving is actually rearranging the data sampling sequence and, thus, can make data loss more random. Therefore, after interleaving, the packet length effect on mutual coherence is almost eliminated, and with the same M/N, their mutual coherence is almost the same.

Figure 24b further shows interleaving improvement on the recovery error with different packet lengths. When M/N is the same, a shorter packet length generally can achieve a lower recovery error, and once its measurement number exceeds the threshold required for accurate recovery (Equation (3)), its recovery error diminishes drastically. While interleaving eliminates the packet length effect, the trend of recovery error with varying M/N is almost the same.

## 8. Conclusions

In this paper, we propose a CS-based framework for sparse signal transmission to solve the bottleneck of expensive lossy link utilization in the transitional region. Specifically, the lossy link transmission is modeled as a random compressive sampling process. Then, the original signal is reconstructed based on the correctly-received packets using CS reconstruction algorithms. Moreover, the packet length effect on signal/information transmission is investigated, which enlightens us to choose different optimal packet lengths to optimize different communication demands.

Extensive simulations have been conducted, and the results show that compared to traditional link-layer automatic repeat request (ARQ) interpolation technique, the proposed method delivers a higher quality reconstructed signal while significantly reducing energy consumption at the transmitter side. Moreover, the packet length effect on signal transmission efficiency can be quite different compared to data transmission efficiency, which is influenced by the specific application and channel condition.

Further experiments using STM32W108 chips conforming to the IEEE 802.15.4 standard verify the sparse signal transmission performance and packet length effect on sparse signal transmission. Interleaving’s improvement is also investigated to validate its performance on enlarging optimal packet length.

Our future work will concentrate on utilizing the link’s characteristics to enhance our CS-based method’s performance on medium access control and routing. Furthermore, the combination of source coding and channel coding will be further studied.
